# Association of NF-E2 Related Factor 2 (Nrf2) and inflammatory cytokines in recent onset Type 2 Diabetes Mellitus

**DOI:** 10.1038/s41598-018-22913-6

**Published:** 2018-03-23

**Authors:** Dornadula Sireesh, Umapathy Dhamodharan, Krishnamoorthy Ezhilarasi, Viswanathan Vijay, Kunka Mohanram Ramkumar

**Affiliations:** 1 SRM Research Institute, SRM Institute of Science and Technology, Kattankulathur-603 203, Tamilnadu, India; 2 Department of Biotechnology, School of Bioengineering, SRM Institute of Science and Technology, Kattankulathur-603 203, Tamilnadu, India; 3 Department of Biochemistry and Molecular Genetics, Prof. M. Viswanathan Diabetes Research Centre and M.V. Hospital for Diabetes (A WHO Collaborating Centre for Research, Education & Training in Diabetes), International Diabetes Federation, Centre of Education and Centre of Excellence in Diabetes Care, Royapuram, Chennai, 600 013 India

## Abstract

We investigated the association of redox regulator Nuclear factor erythroid 2-related factor 2 (Nrf2) and inflammatory cytokines as well as clinical remission in patients with recent onset type 2 diabetes (DM). Blood was collected from 180 DM patients (105 males/75 females) and 150 control subjects (86 males/64 females). Blood glucose, HbA1c, lipid profile and Nrf2 levels were determined along with circulatory cytokines in study subjects. The data were adjusted with confounding factors such as age and sex using multiple logistic regression analysis. We found that Th1/Th2 and oxidative stress markers were significantly elevated, whereas Nrf2 and its downstream targets were decreased in peripheral blood mononuclear cells (PBMCs) of DM subjects when compared with control. The circulatory levels of Nrf2 showed a positive correlation with Th2 cytokines and negative correlation to Th1 cytokines. Further, the impaired insulin secretion in pancreatic β-cells observed due to cytokine stress has been restored by activation of Nrf2 as assessed by glucose-stimulated insulin secretion (GSIS). This study identifies Nrf2 plays a central role in skewing Th1 and Th2 dominance in the progression of diabetes.

## Introduction

Diabetes Mellitus is a pandemic disorder mainly characterized by hyperglycemia due to defect in insulin secretion, insulin action or both. According to International Diabetes Federation (IDF), globally 415 million people have been diagnosed with diabetes in 2015 and expected to rise to 642 million by 2040^1^. Several cross-sectional and prospective studies have been demonstrated that oxidative stress^2^, T-cell and macrophage-derived cytokines^3^ and genetic factors^4^ are important contributors for diabetes and its associated complications. But the precise molecular mechanism is not fully explored.

Oxidative stress plays a pivotal role in the progression of diabetes that contributes in the oxidative modifications of cellular proteins also results in the development of insulin resistance, pancreatic β-cell dysfunction, impaired glucose tolerance and mitochondrial dysfunction^5,6^. It is a known fact that people with diabetes were reported to be vulnerable to oxidative stress due to low levels of the antioxidant defensive mechanism^7^. In addition to oxidative stress, T-cell derived cytokines have also found to be involved in the immune mediated devastation of pancreatic β-cells which results in an imbalance in glucose homeostasis and insulin resistance^8^. In particular, T helper cells1 (Th1) cytokines such as TGF-β, IFN-γ, GM-CSF and IL-2 are mainly involved in inflammatory immune responses, on the other hand, T helper cells2 (Th2) cytokines such as IL-4, IL-5, IL-10 and IL-13 are reported to have anti-inflammatory properties^3^. Based on the available literature, it has been reported that altered balance between T helper type 1 and 2 (Th1 and Th2) cells play a key role in pathogenesis of DM^3,9^.

Nuclear factor erythroid 2-related factor 2 (NFE2L2/Nrf2) a member of CNC (cap ‘n’ collar) subfamily of the basic leucine zipper transcription factor, acts as a master regulator of the redox homeostasis^10^. Under physiological conditions, Nrf2 is associated with its negative regulator, Kelch-like ECH-associated protein-1 (Keap1), whereas in response to stress, this complex dissociates and Nrf2 translocate to the nucleus where it binds to antioxidant responsive element (ARE) and facilitates a multiple functions including, antioxidant activity, detoxification, maintenance of cellular redox homeostasis, glutathione homeostasis and influences mitochondrial biogenesis^11^, by triggering a array of genes, such as Heme Oxygenase-1 (HO-1), Superoxide Dismutase (SOD), NAD(P)H Quinone Oxidoreductase 1 (NQO1) and Glutathione S-transferase (GST)^10,12^.

Recent evidence has shown that Nrf2 has been reported as a promising therapeutic target for various diseases^13^. In particular with diabetes, Nrf2 activation protects pancreatic β-cells against various insults thereby maintain glucose homeostasis and also increase the insulin sensitivity^12^. Earlier studies from our laboratory using *in vitro*, *in vivo* and *in silico* models, we have demonstrated that Nrf2 activation by pterostilbene attenuates both oxidative and pro-inflammatory cytokine toxicity during hyperglycemia through Nrf2 signaling cascade^10,14–16^. Several Nrf2 activators such as resveratrol^17^, sulforaphane^18^, curcumin^19^, quercetin^20^, tert-Butylhydroquinone (tBHQ)^21^ and CDDO (2-cyano-3,12-dioxooleana-1,9(11)-dien-28-oic acid)^22^ have shown to protect pancreatic β-cells and its functions against oxidative and inflammatory stress-induced apoptosis.

In the present study, we investigated the circulatory levels of Nrf2 along with its downstream targets among newly diagnosed type 2 diabetes mellitus (DM) subjects and compared with healthy controls. Further the association of Nrf2 with inflammatory cytokines has been investigated. In addition, the role of Nrf2 activation on insulin secretion against cytokine stress in pancreatic β-cells has been studied.

## Materials and Methods

### Study design and population

In this cross-sectional study, we recruited a total of 330 subjects, divided into two groups: Healthy Control subjects as Group-I (n = 150; M86, F64) and subjects with newly diagnosed DM as Group-II (n = 180; M105, F75). Diagnosis of both control and DM were described earlier from our laboratory based on WHO classification^23^. In brief, healthy control refers to subjects with no history of DM and having fasting plasma glucose (FPG) < 5.6 mmol/l and 2 hr postprandial plasma glucose (PPG) value ≤ 7.8 mmol/l (140 mg/dl) during an oral glucose tolerance test^24^. On the other hand, DM subjects had FPG level of ≥7.0 mmol/l (≥126 mg/dl) and/or PPG level of ≥11.1 mmol/l (≥200 mg/dl)^24^; and all the study samples were collected from outpatient department of M.V. Hospital for Diabetes, Royapuram, Chennai, India. Newly diagnosed DM subjects were particularly recruited for this study to eliminate the confounding effect of medication with the circulatory cytokines, and all those subjects were not previously exposed to any anti-diabetic drugs. The study protocol was approved by the institutional ethics committee (Ref # IEC/N-007/09/2016) and all methods were performed in accordance with the relevant guidelines and regulations of the institution. The written informed consent was obtained from all study participants. The inclusion criteria were adult subjects within the normal range of white blood cells to minimize the confounding effect of infections. The exclusion criteria include subjects with the history of diabetic ketoacidosis or hypoglycemic coma in the past 3 months preceding the study, who received anti-diabetic drugs, anti-inflammatory drugs, anti-hypertensive drugs, statins and other types of diabetic subjects including gestational diabetes, Type 1 DM were also excluded from this study.

### Sample Collection and Biochemical Analysis

Anthropometric measurements, such as height, weight and body mass index were performed by standardized techniques. The fasting blood samples were obtained from the study subjects and was collected in different tubes for plasma and peripheral blood mononuclear cell (PBMC) [EDTA coated Vacutainer tubes], and for serum [Serum-separating tube]. Samples were stored at −80°C for no longer than 9 months, with an average of 5 months and thawed only once. Various biochemical parameters such as fasting blood glucose (FPG) and post prandial glucose (PPG), HbA1c, serum lipid profile including, high and low density lipoprotein cholesterol (HDL-c and LDL-c) were measured using Hitachi-912 Auto analyzer (Hitachi, Mannheim, Germany) using commercial kits (Roche Diagnostics, Mannheim, Germany).

### Sample size calculation and power of study

A pilot study was first carried out using 50 subjects per group. Based on these preliminary results, with a confidence interval of 95%, an estimated *p* value < 0.05, and a power of 80%, the present sample size was derived.

### MDA assay

MDA was measured in serum samples of both control and DM subjects as per the manufacturer’s protocol (Cayman chemicals, MI., USA). Briefly, serum samples were boiled for one hour along with sodium dodecyl sulfate and freshly prepared TBA color reagent. After boiling, samples mixture was immediately place in ice-bath for 10 min, followed by centrifugation at 1600 g for 10 min at 4 °C, and supernatant was used for colorimetric analysis.

### Circulatory Nrf2 levels

The circulatory levels of plasma Nrf2 were measured using Human Nuclear factor erythroid 2-related factor 2 (NFE2L2) ELISA kit, as per the manufacturer’s protocol (Cusabio, MD, USA). Briefly, samples along with the provided standards were incubated for 2 hr in pre-coated 96-well plate followed by Biotin-antibody incubation for 1 hr. After incubation all the wells were washed and incubated with HRP-avidin for 1 hr followed by TMB substrate for 15 min in the dark. The reaction was stopped by addition of stop solution and plate was read at 450 nm with wavelength correction at 540 nm using microplate reader (Infinite 1000, Tecan, Switzerland).

### Th1 and Th2 Cytokine Profiling

The profiling of Th1 and Th2 cytokines was carried out using a Human Cytokine Th1/Th2 Assay 9-plex panel in a multiplex bead-based assay system (Bio-Rad, CA, USA). The assay was performed according to the on manufacturer’s protocol using Bio-Plex 200 system based on Luminex technology. Briefly, the plasma samples were transferred to magnetic beads and incubated for 1 hr at room temperature. After incubation, a series of steps including plate wash, antibody and streptavindin incubation before exposure of plate. Finally, the samples were acquired via cytometric imaging using Luminex xMAP analyzer (Luminex 100 Milliplex Analyzer, Luminex Corp. USA) and analyzed using Bio-Plex Manager™ software 6.1 (Bio-Rad, CA, USA). Cytokine concentrations were determined from standard curves prepared on each plate and expressed as picogram per milliliter (pg/ml). The list of cytokines and its detection limits are represented in Table S1.

### Pancreatic β-cells (MIN6) culture and treatment

MIN6 cells (Insulinoma cell line) were cultured in DMEM (Dulbecco’s modified Eagle’s medium) with 10% FBS (fetal bovine serum), 100 U/ml penicillin and 100 μg/ml streptomycin at 37 °C with 5% CO_2_. All experiments were carried out at passages 16–21 at a confluence rate of 70–80%. In order to confirm the key role of Nrf2 on insulin secretion, cells were seeded in a 6-well culture plate and exposed with well-known Nrf2 activator, resveratrol (8 µM) and cytokine cocktail [IL-1β (50-U/mL), TNF-α (1000-U/mL) and IFN-γ (750-U/mL), R&D Systems, Canada], and combination of both Nrf2 activator and cytokines for 24 hr. After treatment conditions, the cells were harvested, lysed and assessed for Nrf2 activation.

### Nuclear and cytosolic fractionation

To assess the Nrf2 activation potential, nuclear and cytoplasmic fractions were separated using Nuclear Extraction Kit (Abcam, UK), according to the manufacturer’s instructions. Briefly, cells were trypsinized after treatment, centrifuged for 5 min at 1000 rpm; the pellet was resuspended in cytosolic-extraction buffer and vortexed vigorously, and the lysate was centrifuged for 2 min at 12,000 rpm at 4 °C; cytoplasmic protein fraction was collected. To the nuclear pellet, extraction buffer with protease inhibitor cocktail and DTT was added and incubated on ice for 15 min. The lysate was centrifuged, and the supernatant containing the solubilized nuclear proteins were collected and used for immunoblot analysis.

### Western blotting

The protein samples extracted form study subjects were subjected to immunoblotting. In brief, protein concentration was estimated using Bradford reagent (Biorad, PA, USA) and denatured with sample buffer, separated on a SDS-PAGE followed by electroblotted onto the nitrocellulose membrane. The membrane was then exposed with blocking solution, followed by overnight incubation at 4°C with primary antibodies (Nrf2, LaminB and β-actin, Abcam, MA, USA). After incubation, the membrane was washed thrice with TBST and probed with an HRP-conjugated secondary antibody. By using an enhanced chemiluminescence (ECL) kit (Biorad, PA, USA), the signals were detected and captured using documentation system (GBOX, Syngene, UK).

### RT-PCR analysis

 mRNA was isolated from the PBMCs of study subjects using RNA isolation kit (Qiagen, CA, USA) and an equal quantity of RNA was converted to cDNA via cDNA conversion kit as per the manufacturer’s instructions (Qiagen, CA, USA). The resulted cDNA was used for the expression studies using RT-PCR (Biorad CFX connect systems, Biorad, PA, USA). The primer sequences for the target genes are given in the Table S2.

### Glucose-stimulated insulin secretion (GSIS)

Pancreatic β-cells (MIN6) were cultured in 96-well plate followed by Nrf2 activator and cytokine exposure. After treatment, the cells were incubated in Krebs–Ringer bicarbonate buffer (KRBB) with low glucose (2.8 mM) for 1 hr at 37 °C, which allowed building up of insulin in cells. After 1 hr, incubation medium was changed to fresh KRBB containing either basal (2.8-mM, basal insulin release) or stimulatory (28-mM, stimulated insulin release) glucose concentrations, and extended incubation for an additional 1hr at 37 °C. Supernatants were collected for insulin measurements using mouse-specific insulin ELISA kit (Mercodia, Uppsala, Sweden). Data were normalized with protein content determined by Bradford reagent (Bio-Rad, Hercules, CA, USA). Assays were performed in three independent experiments.

### Statistical analysis

Statistical calculations were performed using SPSS (version 20.0, Chicago, IL). Normally distributed data are represented as mean ± SEM. Student’s “t” test and Mann-Whitney “U” test were used to determine the statistical significance. Multivariate logistic regression analysis was used to determine the association of cytokines with the DM subjects. Correlation analysis was performed using Pearson correlation analysis in SPSS.

## Results

### Clinical and biochemical characteristics of the study subjects

Selected clinical and biochemical characteristics of study subjects are presented in Table 1. Compared to control, the DM subjects were significantly older in age (*p* < 0.0001), had higher SBP (*p* < 0.0001), DBP (*p* < 0.0001), FPG (*p* < 0.0001), PPG (*p* < 0.0001), HbA1c (*p* < 0.0001), total serum cholesterol (*p* < 0.0001) and LDL-c (*p* < 0.0002). However, both Body Mass Index (BMI) and HDL-c were found to be non-significant in DM subjects, when compared with healthy controls.

**Table 1:** Clinical and biochemical characteristics of the study subjects.

Clinical parameters	Control (n = 150)	DM (n = 180)	*P*
M:F	86:64	105:75	—
Age (Years)	40.64 ± 7.1	48.1 ± 10.41	**0**.**0001**
BMI (Kg/m^2^)	25.17 ± 0.2.35	25.73 ± 3.51	0.0964
SBP (mm Hg)	101.9 ± 11.5	121.8 ± 15.4	**0**.**0001**
DBP (mm Hg)	77.5 ± 7.3	82.9 ± 9.3	**0**.**0001**
FPG (mg/dL)	93.8 ± 8.4	165.1 ± 54.84	**0**.**0001**
PPG (mg/dL)	117.2 ± 16.1	254.8 ± 51.49	**0**.**0001**
HbA1c (%)	5.12 ± 0.2	8.4 ± 2.47	**0**.**0001**
Total serum Cholesterol (mg/dL)	141.6 ± 30.11	179.4 ± 41.78	**0**.**0001**
HDL-cholesterol (mg/dL)	40.51 ± 8.47	41.8 ± 6.58	0.1206
LDL-cholesterol (mg/dL)	101.7 ± 19.46	112.98 ± 31.71	**0**.**0002**

BMI- Body mass index; SBP-Systolic Blood Pressure; DBP-Diastolic Blood Pressure; FPG-Fasting plasma glucose; PPG-Postprandial plasma glucose; HbA1c- Glycated haemoglobin; HDL- High Density Lipoprotein; LDL-Low Density Lipoprotein. *p*-values were calculated using student’s “t” test and Mann-Whitney “U” test on SPSS software.

### RT-PCR analysis of oxidative stress markers

**Figure 1:**
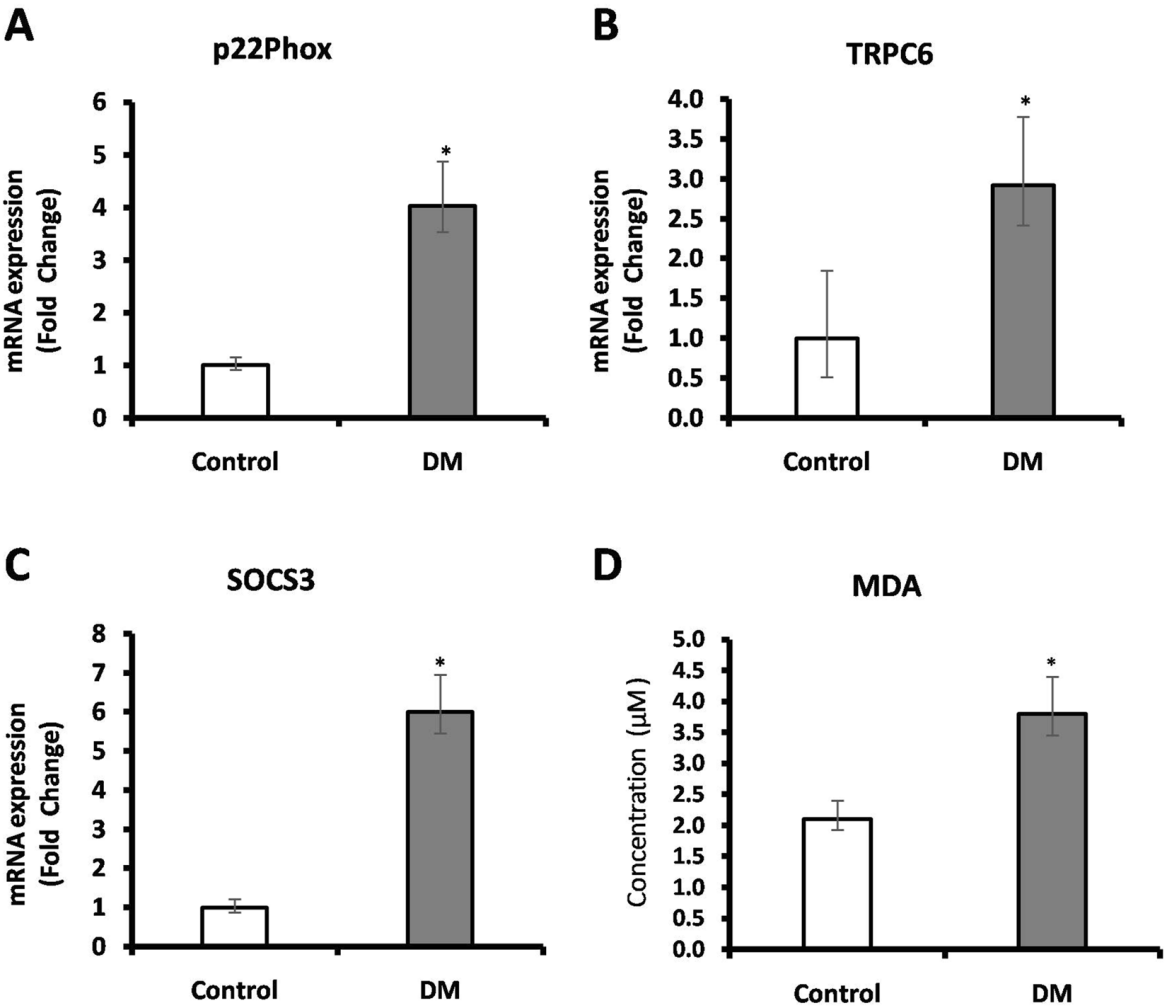
The expression of selected oxidative stress markers in PBMC of study subjects were measured using RT-PCR. Transcript levels of (**A**) p22phox (**B**) TRPC6 (**C**) SOCS3 and (**D**) Serum MDA levels of study subjects. Data are expressed as fold change over control and presented as mean ± SEM of three independent experiments. Statistical analysis was performed by students ‘t’ test. *p* < 0.05.

Figure 1 depicts the expression of selected oxidative stress markers in PBMC of study subjects. In DM subjects, the expression of p22^phox^, TRPC6 and SOCS3 were found to be increased up to 4.03-fold (*p* < 0.005), 2.92-fold (*p* < 0.004) and 6.00-fold (*p* < 0.004) respectively, when compared to control subjects. Also, the serum levels of MDA were measured in study subjects which showed 1.81-fold (*p* < 0.0001) increase in DM with respect to control subjects.

### Levels of Nrf2 and its downstream target genes

**Figure 2:**
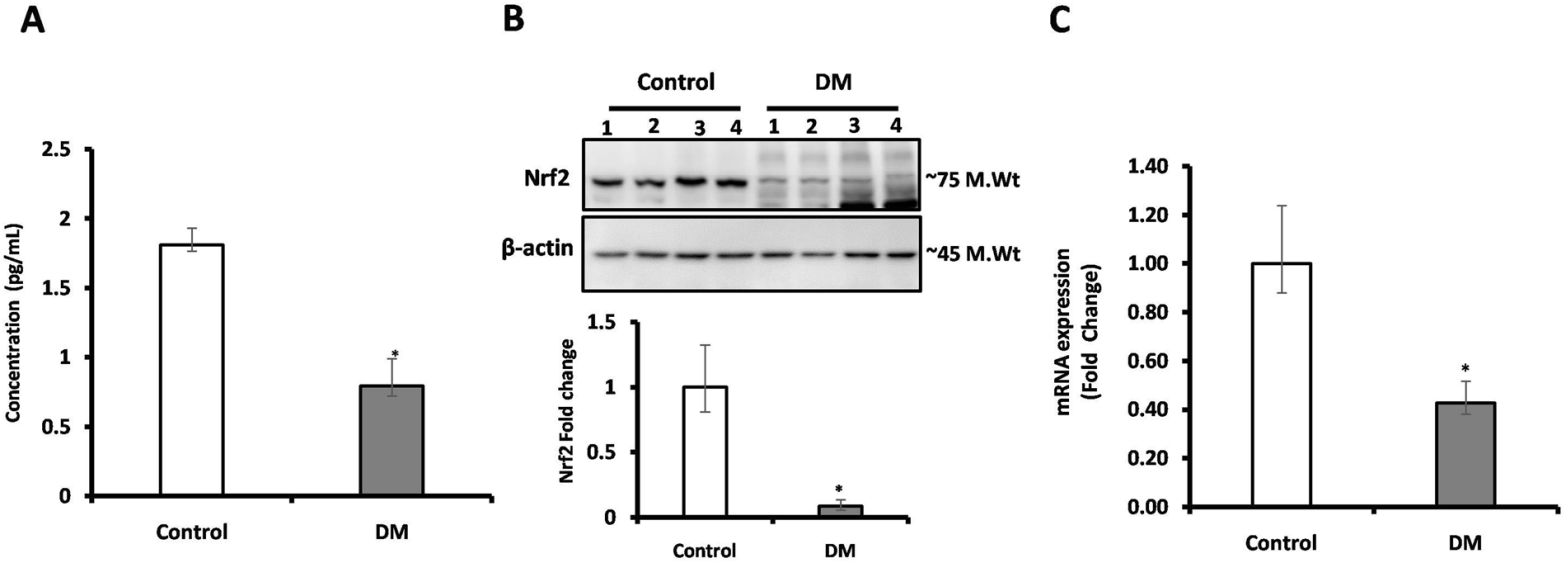
(**A**) The circulatory level of Nrf2 was measured using ELISA, (**B**) protein expression of Nrf2 was measured in PBMC of study subjects using western blot (**C**) mRNA levels of Nrf2 was measured in PBMC of study subjects using RT-PCR. Data are presented as mean ± SEM of three independent experiments. Statistical analysis was performed by students ‘t’ test.  *p *< 0.05.

**Figure 3:**
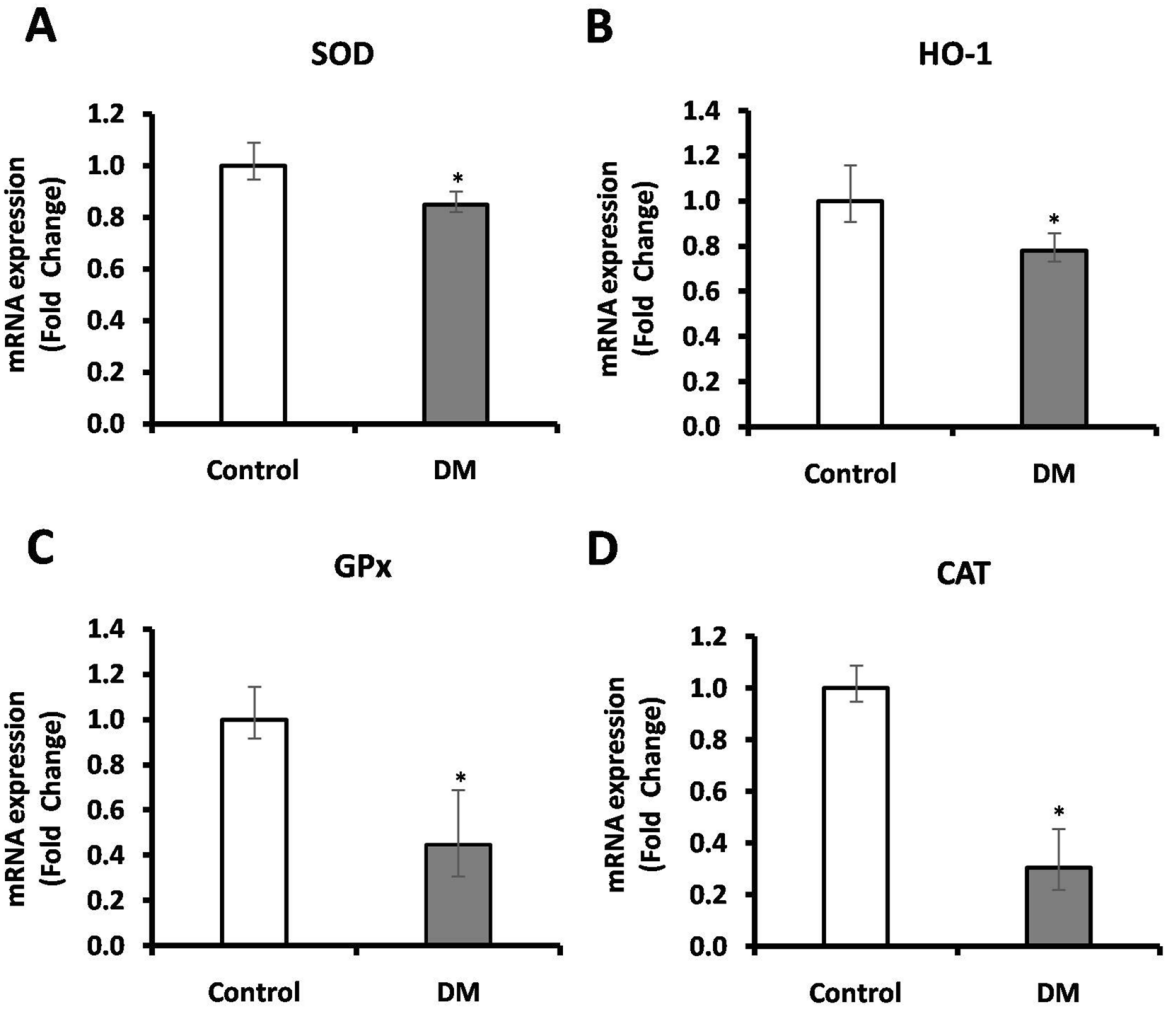
The levels of Nrf2 downstream genes such as (**A**) SOD, (**B**) HO-1, (**C**) GPx and (**D**) CAT in PBMC of study subjects were measured using RT-PCR analysis. Data are expressed as fold change over control and presented as mean ± SEM of three independent experiments. Statistical analysis was performed by students ‘t’ test.  *p *< 0.05.

The gene expression, protein expression and circulatory levels of Nrf2 was assessed using RT-PCR, western blot and ELISA respectively. Plasma Nrf2 levels were found to be lowered (0.79 pg/mL) in DM subjects when compared to control subjects (1.8 pg/mL) (Fig. 2A). Further, to measure the Nrf2 protein expression in PBMC levels of study subjects, total protein was extracted and subjected to western blot and expressed as fold change. We found 0.43-fold lower level expression of Nrf2 in PBMC of DM compared with control subjects (Fig. 2B, S.Fig. 1). Further, we measured mRNA expression of Nrf2 and its downstream target genes in PBMC of study subjects via RT-PCR. In DM subjects the mRNA expression of Nrf2 (0.43-fold, *p* < 0.02) (Fig. 2C), SOD (0.85-fold, *p* < 0.01) (Fig. 3A), HO-1 (0.78-fold, *p* < 0.01) (Fig. 3B), GPx (0.45, *p* < 0.004) (Fig. 3C) and CAT (0.31-fold, *p* < 0.03) (Fig. 3D) showed low levels with respect to control subjects.

### Levels of Th1 and Th2 cytokines in study subjects

Human cytokine Th1/Th2 9-plex assay panel with Th1 cytokines (IFN-γ, IL-2, IL-12, TNF-α, GM-CSF) and Th2 (IL-4, IL-5, IL-10, IL-13), was used to measure the circulatory cytokines in study subjects. Out of 9 inflammatory cytokines analyzed, six-cytokines including IL-4 (*p* = 0.0317), IL-10 (*p* = 0.0159), IL-13 (*p* = 0.0317), IFN-γ (*p* = 0.0286), GM-CSF (*p* = 0.0357) and TNF-α (*p* = 0.0357) were found to be significantly increased, whereas IL-2, IL-5 and IL-12 did not show significant difference in DM when compared with control subjects (Table 2**)**. Further, we analyzed the hazard ratio for all studied cytokines using multiple logistic regression analysis for the adjustment of age and sex percentiles (Table S3**)**.

**Table 2:** Circulatory levels of inflammatory cytokines in the study subjects as assessed by multiplex assay.

Cytokines	Control	DM
IL2	3.95(4.89–7.78)	4.11(3.97–8.47)
IL-4	2.08(0.75–5.75)	8.52 (5.22–14.47)*
IL-5	23.71(9.18–27.06)	42.75(20.94–69.93)
IL10	19.680(2.885–28.66)	72.15(44.79–102.2)*
IL12	5.923(2.23–8.725)	8.1(5.87–36.44)
IL-13	4.545(2.95–12.06)	15.11(10.44–19.37)*
IFN-γ	112.8(75.66–179.6)	325.9(183.8–473.4)*
GM-CSF	4.21(2.94–8.45)	23.88(12.94–43.59)*
TNFα	35.12(10.73–54.08)	98.25(58.63–138.8)*

IL, interleukin; IFN, interferon; TNF, tumor necrosis factor; GM-CSF, Granulocyte-macrophage colony-stimulating factor. All data are reported as median (range). *Indicates DM subjects compared with control subjects. *p < 0.05.

### Association between Nrf2 with Th1 and Th2 cytokines in study subjects

**Figure 4:**
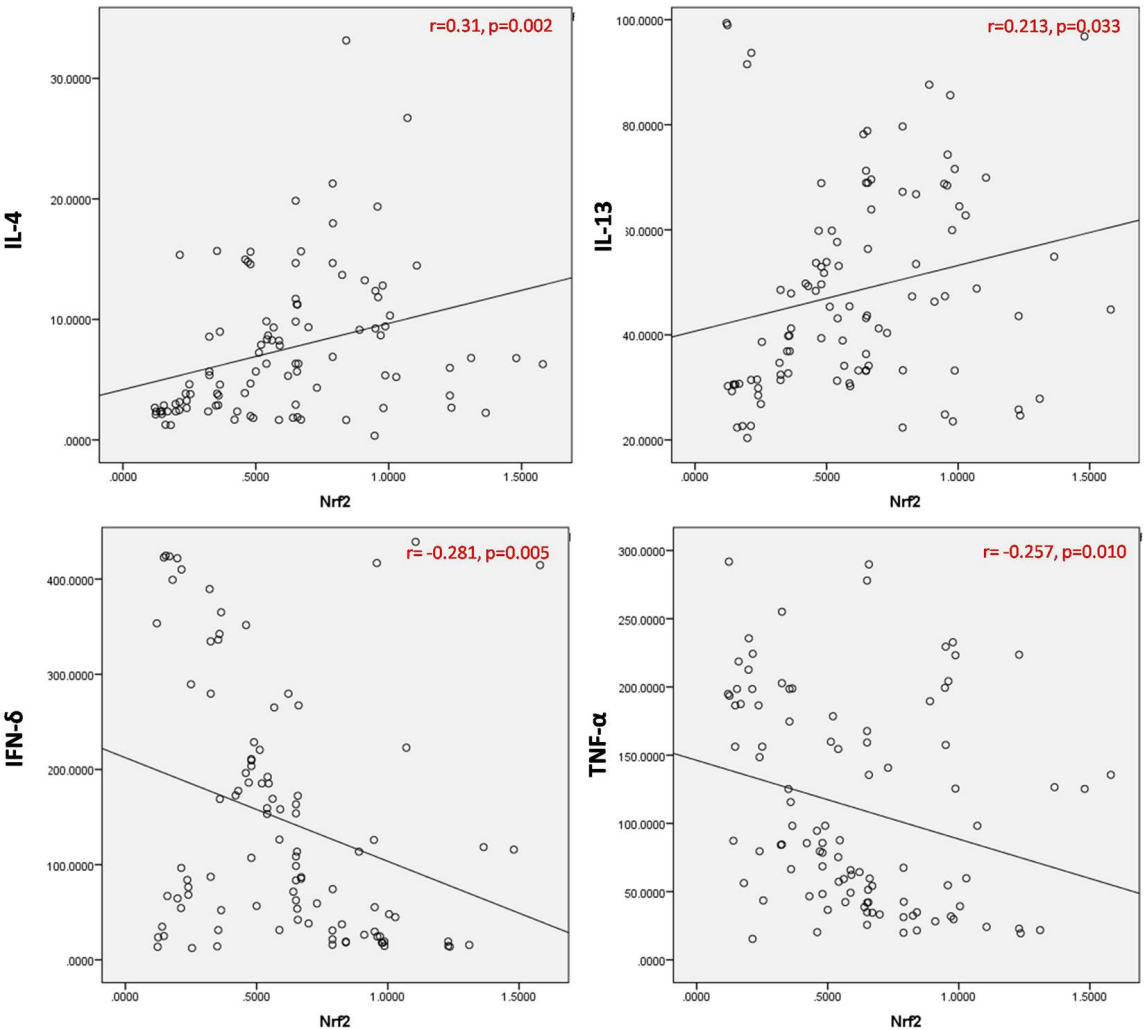
Pearson correlation analysis between Nrf2 and Th1/Th2 cytokines in plasma among the study subjects. Plasma levels of Nrf2 were positively correlated with Th2 cytokines (IL-4 & IL-13) and negatively correlated with Th1 cytokines (IFN-γ & TNF-α).

We further correlated the levels of Nrf2 alterations with Th1 and Th2 signatures in DM subjects. Pearson correlation showed a strong positive correlation of Nrf2 with Th2 cytokines such as IL-4 (r = 0.31;  *p* = 0.002), and IL-13 (r = 0.213;  *p* = 0.033) and a strong negative correlation with Th1 cytokines [IFN-γ (r = -0.281;  *p* = 0.005), TNF-α (r = -0.257;  *p* = 0.01)] (Fig. 4).

### Pearson’s correlation between Nrf2 and clinical parameters of study subjects

We further correlated the mean levels of Nrf2 with clinical parameters in DM subjects. Pearson’s correlation showed a strong negative correlation of Nrf2 with age (r = −0.170; *p* = 0.019), FPG (r = −0.167; *p* = 0.012), HbA1c (r = −0.199; *p* = 0.021), total serum cholesterol (r = −0.225; *p* = 0.025) and other parameters were analyzed and represented in the Table S4.

### Effect of Nrf2 activation on insulin secretion against cytokines stress in pancreatic β-cells

**Figure 5:**
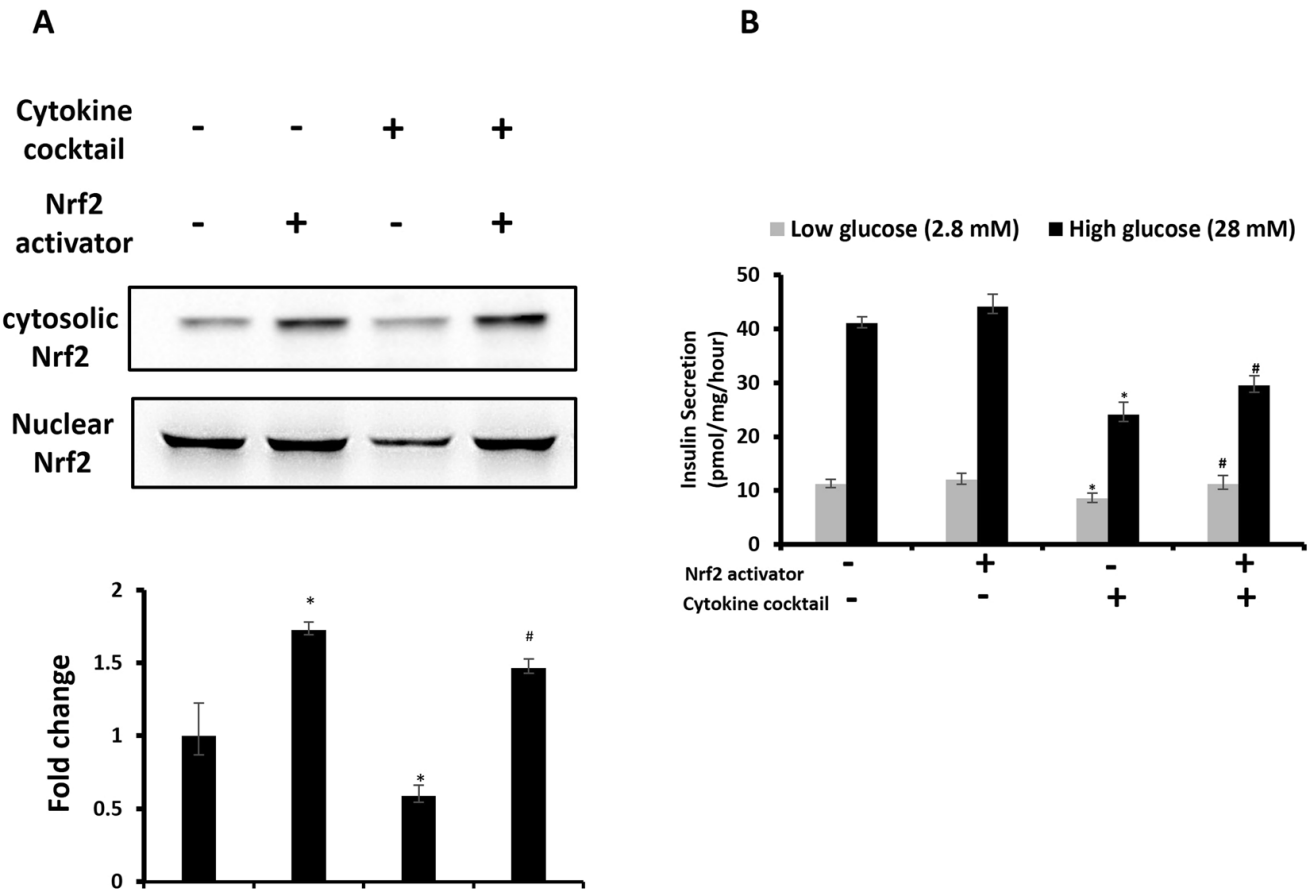
(**A**) Effect of Nrf2 activator, resveratrol on nuclear translocation of Nrf2 in cytokine cocktail-treated pancreatic β-cells. (**B**) Glucose-stimulated insulin secretion assay were performed in Krebs-Ringer bicarbonate buffer containing low (2.8 mM) and high (28 mM) glucose concentrations to study the effect of Nrf2 activator on insulin secretion in cytokine exposed cells. Data are expressed as mean ± SEM of three separate experiments. Statistical analysis was performed using one way ANOVA.  *p *< 0.05.

In order to study the effect of Nrf2 activation on insulin secretion against cytokine stress, we exposed insulin secreting pancreatic β-cells, MIN6 with Nrf2 activator, resveratrol and cytokine cocktail, and assessed for Nrf2 expression as well as glucose stimulated insulin secretion (GSIS). Immunoblot analysis revealed that, resveratrol treatment significantly increase (*p* < 0.05) the levels of Nrf2 protein in the nuclear extract with an associated decrease in cytoplasmic extract of untreated and cytokine cocktail-treated cells (Fig. 5A, S.Fig. 2).

To study the effect of Nrf2 activation on insulin secretion against cytokine stress, after the experimental conditions, cells were subjected to 1 hr static incubations in krebs-ringer bicarbonate buffer containing basal (2.8 mM) and stimulatory (28 mM) glucose. There was a significant drop in insulin level in cytokine-exposed cells when compared with the control. Moreover, Nrf2 activator, improved the insulin secretion in response to static glucose incubation and cytokine stress (*p* < 0.05) (Fig. 5B). These results provide evidence that Nrf2 activation improves insulin secretion against cytokine stress in pancreatic β-cells.

## Discussion

Several pathogenic processes are involved in the development of diabetes, range from autoimmune destruction of the pancreatic β-cells with consequent insulin deficiency to abnormalities that result in resistance to insulin action. It has been well documented that several factors including oxidative stress, pro-inflammatory stress, nitroso stress, endoplasmic reticulum stress and environmental factors are involved in the progression of diabetes^10,12,25,26^. Among those factors, hyperglycemia mediated oxidative stress stands as a base for the development of various complications of DM which is evidenced by an elevated level of reactive oxygen species (ROS) due to tampered levels of antioxidant genes^10,12^. On the other hand, ROS is also reported to act as a signaling molecule and mediator for inflammation^2^. Hence, there is an imperative need to understand the defense mechanism to overcome various stressful stimuli. Nrf2 is a master regulator of various antioxidants and anti-inflammatory cytokines by triggering its downstream targets genes. Based on the recent studies, it has been reported that Nrf2 is a promising therapeutic target for diabetes and its late complications^12,27,28^. However, under clinical settings to the best of our knowledge, this is the first line of evidence to explain the correlation of circulatory levels of Nrf2 with both oxidative stress and inflammatory cytokines in DM subjects.

Our results on the gene expression of selective oxidative stress markers such as TRPC6, p22^phox^, SOCS3 showed a significant increase in subjects with DM. Recently, Tilo *et al*., reported TRPC6 mRNA expression was elevated in high glucose induced oxidative stress in human monocytes^29^. Accumulating evidence on TRPC6 indicates its cellular functions by acting as a receptor for calcium entry, stabilizing cell morphology and maintaining the internal structure of a cell and also serves as a downstream effector of ROS generation^29,30^. Further several researchers targeted oxidative stress during hyperglycemia due to its various sources of intracellular ROS generation in which NADPH oxidase emphasized as a major source for superoxide and hydrogen peroxide generation^31^. Hence, NADPH oxidase subunit p22^phox^ was measured and was found to be elevated in DM subjects. In line with the preceding reports, p22^phox^ gene expression was significantly higher in leucocytes of DM compared with control subjects^31^. The results of the present study were found to be in congruence with these aforementioned reports. SOCS family proteins are classical proteins that are induced by various cytokines which are involved in insulin resistance^32^. Studies have shown that during hyperglycemia, SOCS3 expression was significantly up-regulated in DM subjects compared with control^32^. In addition to hyperglycemia, TNF-α (Th1 cytokine) also reported to directly induce SOCS3 expression^32^. In the present study, both SOCS3 and TNF-α were found to be significantly increased in DM when compared with control subjects. These results are supportive with an earlier finding by Jennifer *et al*., showed that elevated levels TNF-α and IL-6 levels in DM subjects induce SOCS3 expression^32^. Furthermore, we measured MDA levels, which is again an indicator of oxidative stress as determined in various disease models. Results of the present study showed MDA levels were significantly elevated in DM subjects as compared to control subjects. Several studies emphasized that the elevated levels of MDA were found in DM subjects^31,33^. Our findings are in agreement with Shiny *et al*., from the similar population which has shown the MDA levels were significantly increased in DM subjects^33^. Taken together, these results suggest that oxidative stress is one of the predominant markers in DM subjects.

Th1 and Th2 cytokine balance plays a key role in upholding of normal immune response. Association of Th1 cytokines has been reported to be involved in the destruction of pancreatic β-cells through the release of cytotoxic mediators such as nitric oxide, oxygen radicals, serine esterases, etc^34,35^. Few other reports also highlighted the importance of Nrf2 activators in skewing of Th2 mediated immunity but the exact mechanism is still unclear^3^. Overall these studies highlighted that Nrf2 signaling showed a regulatory mechanism in Th1 and Th2 mediated immunity. Several reports stated that Nrf2 activation inhibits Th1 cytokine production by promoting Th2 cytokine production^36,37^. Additionally, studies also evidenced that, Nrf2 activator tBHQ promotes transcriptional activity of Th2 cytokines (IL-4, IL-5, IL-13) and suppress Th1 cytokine IFN-γ production^36^. Moreover, Nrf2 deficient dendritic cells showed elevated levels of oxidative stress that confers a Th2-like immune responsiveness, which leads to alter in Th1 and Th2 balance^36^. Hence, the present study was designed to investigate the association between the Nrf2 with Th1&Th2 cytokines in DM.

The results of the present study revealed that cytokines such as IFN-γ, TNF-α and GM-CSF (Th1 cytokine family) showed elevated levels in DM as compared with control subjects. These findings were in harmony with few earlier reports^38,39^. On the other hand, IL-2 (Th1 cytokine) didn’t show any significant difference between the study subjects, interestingly these findings were similar to an earlier finding from Madhumitha *et al*., in which the levels of IL-2 didn’t have any significant difference in DM when compared with control subjects^38^. In the same study, it also shown that, Th2 cytokines namely IL-4 and IL-13 to have a significant difference in DM subjects when compared to healthy controls, with no significant differences in IL-5 levels^38^. However, these aforementioned studies are in well agreement with the present study that, both IL-4 and IL-13 showed significant difference whereas IL-5 didn’t show any difference in study subjects among South Indian population. Further, we found significantly elevated plasma levels of IL-10 in DM subjects when compared to control subjects. This finding of ours is in line with previous reports which have documented that levels of IL-10 found to be increased when compared to control subjects^40^.

In the present study, we found the low level of Nrf2 among DM subjects as compared with control subjects. Our findings coincided with few reports including osorio *et al*., who studied on Mexican population, a significant decrease in the levels of Nrf2 in subjects with DM having uncontrolled blood sugar levels, when compared to subjects having DM with controlled blood sugar^27^. Another study from Chinese population by Xia Wang *et al*., reported that Nrf2 polymorphism (compared individuals with the CC genotype, those with the AA genotype) in DM subjects has significantly associated with decreased Nrf2 and its antioxidant status and also demonstrated that those subjects are highly susceptible to oxidative stress^28^. Moreover, several studies demonstrated that significant decrease in antioxidant genes (HO-1, GPx, SOD, CAT) of DM compared with control subjects^27,28^. In line with these reports, our results also revealed the lower level of Nrf2 downstream antioxidant genes in DM subjects when compared with healthy controls.

Few studies highlighted the role of Nrf2 and its downstream target genes in various pathological conditions^41^. Moreover, several *in vitro* and *in vivo* studies emphasized the anti-diabetic role of Nrf2 activators by attenuating both oxidative and inflammatory stress. Moreover, Nrf2 knockout mice are found to be highly prone to oxidative and inflammatory insults^42,43^. Few studies demonstrated that, insulin secretion was inhibited in pancreatic β-cells due to cytokine and glucose insult and restored by Nrf2 activation^10,44^. These results are consistent with our present findings that, Nrf2 activator showed an impact on insulin secretion process via Nrf2 signaling cascade^10^.

Interestingly, we observed a strong positive correlation between plasma Nrf2 and Th2 cytokine (IL-4, IL-13) levels, meanwhile a strong negative correlation with Th1 cytokine (IFN-γ, TNF-α) levels. Further, Rockwell *et al*., stated that Nrf2 activation by tBHQ in CD4^+^ T cells, inhibits Th1 cytokine production, whereas it was concurrently stimulating the secretion of Th2 cytokines but not in Nrf2 knockout cells^36^. On the other hand, reduced levels of Th2 cytokines and higher levels of Th1 cytokines in progression of metabolic diseases were reported in Kuwait and south Indian populations^38,45^. Compiling the results of the present study, we conclude that Nrf2 plays a central role in skewing Th1 and Th2 dominance in the progression of diabetes. Limitation of the present study is that it is cross sectional in nature, which means that no cause and effect relationship can be drawn from the present study. Although the levels of Nrf2 regulate blood glucose and insulin secretion which was well documented by *in vitro* and *in vivo* studies, further investigation is warranted in clinical research.

To conclude, the plasma Nrf2 levels were significantly low in DM when compared with control subjects. Further, Correlation analysis showed that there was a positive correlation between Nrf2 and Th2 cytoines, and a negative correlation between Nrf2 and Th1 cytokines, hence lower levels of Nrf2 favors Th1 response. However, this study was conducted in a high-risk ethnic population and to the best of our knowledge; this is the first line of evidence on the levels of Nrf2 and its downstream targets in clinical settings among South Indian population.

## Electronic supplementary material

Supplementary file
