# Supplementary material for: Association of NF-E2 Related Factor 2 (Nrf2) and inflammatory cytokines in recent onset Type 2 Diabetes Mellitus
Source: Sci Rep. 2018 Mar 23;8:5126. doi: 10.1038/s41598-018-22913-6 (PMC5865120; doi:10.1038/s41598-018-22913-6)

**Association of NF-E2 Related Factor 2 (Nrf2) and inflammatory cytokines in**

**recent onset Type 2 Diabetes Mellitus**

Dornadula Sireesh^1,2^, Umapathy Dhamodharan^1^, Krishnamoorthy Ezhilarasi^3^, Viswanathan Vijay^3^*, Kunka Mohanram Ramkumar^1,2^*

^1^SRM Research Institute, SRM Institute of Science and Technology, Kattankulathur-603 203, Tamilnadu, India

^2^Department of Biotechnology, School of Bioengineering, SRM Institute of Science and Technology, Kattankulathur-603 203, Tamilnadu, India

^3^Department of Biochemistry and Molecular Genetics, Prof. M. Viswanathan Diabetes Research Centre and M.V. Hospital for Diabetes (A WHO Collaborating Centre for Research, Education & Training in Diabetes), International Diabetes Federation, Centre of Education and Centre of Excellence in Diabetes Care. Royapuram, Chennai-600 013, India

***Corresponding authors:**

Dr. Kunka Mohanram Ramkumar, Tel: +91-99407 37854; Fax: +91-44-2745-23437,

e-mail address: ramkumar.km@res.srmuniv.ac.in

Dr. Vijay Viswanathan, Tel: +91- 44-25954913; Fax: +91-44-2595 4919,

e-mail address: drvijay@mvdiabetes.com

**S.Table-1.** List of study markers and its lower detection limit

| **S.No** | **Inflammatory Markers** | **Lower detection limit (pg/ml)** |
| --- | --- | --- |
| 1 | IL-2 | 0.58 |
| 2 | IL-4 | 0.20 |
| 3 | IL-5 | 0.80 |
| 4 | IL-10 | 3.39 |
| 5 | IL-12 (p70) | 2.35 |
| 6 | IL-13 | 0.36 |
| 7 | GM-CSF | 0.66 |
| 8 | TNF-α | 3.18 |
| 9 | IFN-γ | 0.91 |

IL, interleukin; IFN, interferon; TNF, tumor necrosis factor; GM-CSF, Granulocyte-macrophage colony-stimulating factor.

**S.Table-2.** List of primers and its sequences used in the manuscript

| **S.No** | **Gene** | **Forward** | **Reverse** |
| --- | --- | --- | --- |
| 1 | Nrf2 | TTCAGCCAGCCCAGCACATC | CGTAGCCGAAGAAACCTCATTGTC |
| 2 | SOD | GAAGGTGTGGGGAAGCATTA | ACATTGCCCAAGTCTCCAAC |
| 3 | CAT | TCATGACATTTAATCAGGCA | GTGTCAGGATAGGCAAAAAG |
| 4 | GPX | TTCCCGTGCAACCAGTTTG | TTCACCTCGCACTTCTCGAA |
| 5 | HO-1 | CTCTGAAGTTTAGGCCATTG | AGTTGCTGTAGGGCTTTATG |
| 5 | TRPC6 | TTTGAGGAGGGCAGAACACTTCCT | TATGGCCCTGGAACAGCTCAGAAA |
| 6 | P22^phox^ | TTCACCCAGTGGTACTTTGG | GTCATGTACTTCTGTCCCCAG |
| 7 | SOCS3 | TCGATTCGGGACCAGC | GCGGGAAACTTGCTGT |

**S. Table-3.** List of cytokines adjusted for age and sex using multiple logistic regression analysis

| **S.No** | **Inflammatory Markers** | **Unadjusted OR** | | **Adjusted OR** | |
| --- | --- | --- | --- | --- | --- |
|  |  | **OR (95% C.I)** | **p value** | **OR (95% C.I)** | **p value** |
| 1 | IL-2 | 1.0 (0.98-1.0) | 0.863 | 1.0 (0.99-1.0) | 0.84 |
| 2 | IL-4 | 5.7 (0.16-2.17) | **0.04** | 4.9 (0.14-1.57) | **0.03** |
| 3 | IL-5 | 1.18 (0.92-1.45) | 0.19 | 1.15 (0.88-1.59) | 0.24 |
| 4 | IL-10 | 9.04 (1.0-4.6) | **0.007** | 8.08 (0.9-4.4) | **0.003** |
| 5 | IL-12 (p70) | 1.08 (0.93-1.24) | 0.29 | 1.07 (0.94-1.24) | 0.27 |
| 6 | IL-13 | 3.93 (0.74-5.0) | **0.02** | 3.78 (0.68-4.93) | **0.02** |
| 7 | GM-CSF | 7.29 (0.91-1.6) | **0.001** | 7.24 (0.84-1.5) | **0.001** |
| 8 | TNF-α | 8.67 (0.98-1.1) | **0.002** | 8.59 (0.94-1.12) | **0.002** |
| 9 | IFN- γ | 9.01 (0.99-1.0) | **0.003** | 8.12 (0.95-1.15) | **0.002** |

IL, interleukin; IFN, interferon; TNF, tumor necrosis factor; GM-CSF, Granulocyte-macrophage colony-stimulating factor. Odds-ratio (OR) adjusted for confounding factor (age and sex)

**S. Table-4.** Pearson’s correlation of Nrf2 with other clinical and biochemical parameters

|  | **r** | ***p*** |
| --- | --- | --- |
| AGE | -0.170^*^ | **0.019** |
| BMI | 0.058 | 0.570 |
| SBP | 0.001 | 0.995 |
| DBP | -0.019 | 0.848 |
| FPG | -0.167 | **0.012** |
| PPBS | 0.069 | 0.493 |
| HbA1c | -0.199^*^ | **0.021** |
| Total serum Cholesterol | -0.225^*^ | **0.025** |
| HDL | 0.106 | 0.294 |
| LDL | -0.013 | 0.900 |

BMI- Body mass index; SBP-Systolic Blood Pressure; DBP-Diastolic Blood Pressure; FPG-Fasting plasma glucose; PPG-Postprandial plasma glucose; HbA1c- Glycated haemoglobin; HDL- High Density Lipoprotein; LDL-Low Density Lipoprotein. p and r values were calculated using Pearson’s rank correlation test at 95% confidence intervals (CIs).

**S. Figure 1.** The protein levels of Nrf2 (santa cruz, sc-722; 1:250 dilution) was measured in PBMC of study subjects using western blot. Blots with retain at six band widths above and below (since cropped image showed with specific molecular weight in the main body of the paper).


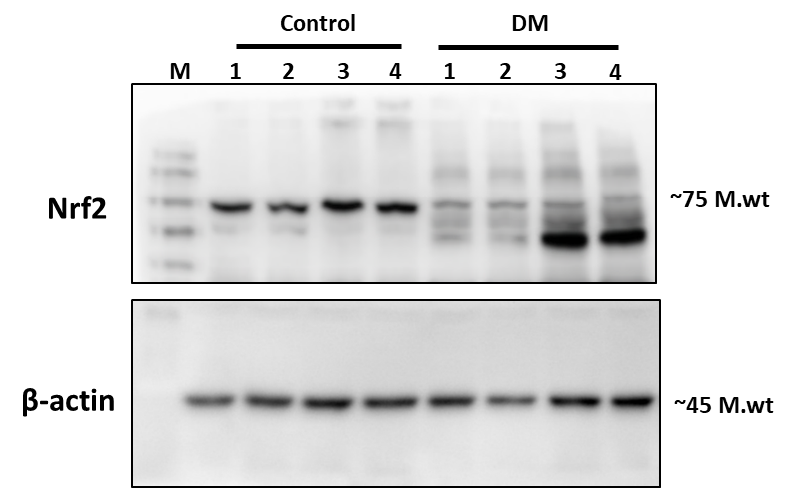


S. F**igure 2.** MIN6 were treated with Nrf2 activator (resveratrol 8μM), cytokine cocktail and combination of Nrf2 activator+cytokine for 24 h. After the treatment, cells were harvested, lysed and assessed for the expression of Nrf2 (santa cruz, sc-722; 1:250 dilution) by Immuno blot. After Nrf2, blots were stripped and checked for respective home-gene expression. Blots with retain at six band widths above and below (since cropped image showed with specific molecular weight in the main body of the paper).


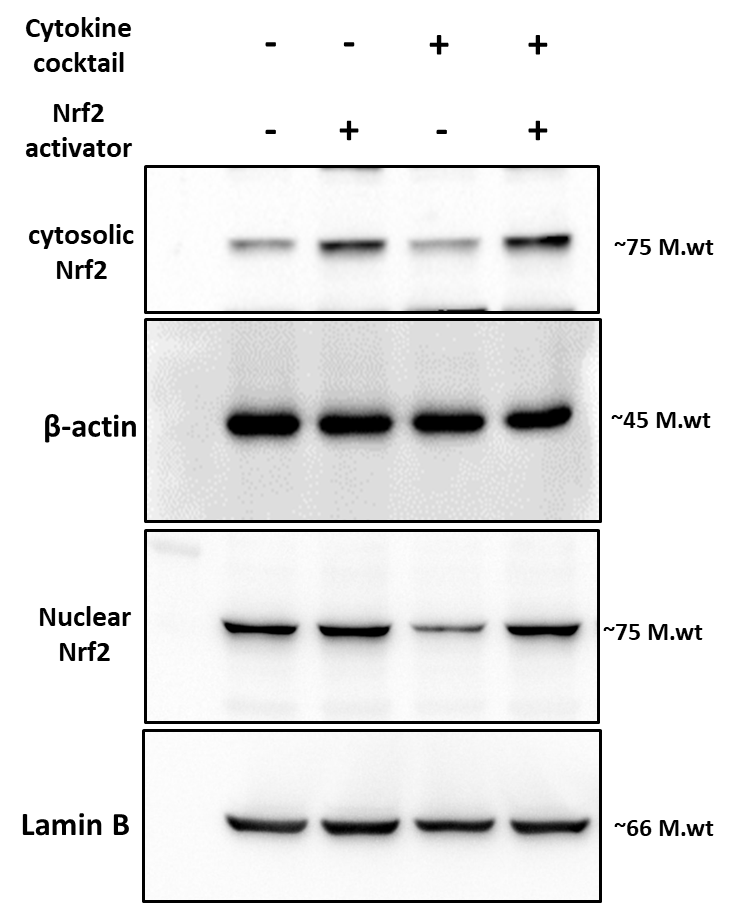

Supplement: Supplementary file 1 — Supplementary file [file 41598_2018_22913_MOESM1_ESM.doc]
